# High Serum Procalcitonin Concentrations in Patients With Hemorrhagic Fever With Renal Syndrome Caused by Hantaan Virus

**DOI:** 10.3389/fcimb.2018.00129

**Published:** 2018-05-07

**Authors:** Xiude Fan, Huan Deng, Jiao Sang, Na Li, Xiaoge Zhang, Qunying Han, Zhengwen Liu

**Affiliations:** Department of Infectious Diseases, First Affiliated Hospital of Xi'an Jiaotong University, Xi'an, China

**Keywords:** hemorrhagic fever with renal syndrome, Hantaan virus, procalcitonin, disease severity, prognosis

## Abstract

**Objective:** This study analyzed the significance of procalcitonin (PCT) in patients with hemorrhagic fever with renal syndrome (HFRS) caused by Hantaan virus.

**Methods:** The demographics and clinical and laboratory data including PCT at hospital admission in 146 adults with HFRS were retrospectively analyzed.

**Results:** PCT level was significantly higher in severe patients (*n* = 72) than in mild patients (*n* = 74, *p* < 0.001) and independently associated with disease severity (OR 2.544, 95% CI 1.330–4.868, *p* = 0.005). PCT had an area under the receiver operating characteristic curve (AUC) value of 0.738 (95% CI 0.657–0.820, *p* < 0.001) for predicting severity. PCT level was significantly increased in patients with bacterial infection (*n* = 87) compared with those without (n = 59, *p* = 0.037) and associated with bacterial infection (OR 1.685, 95% CI 1.026–2.768, *p* = 0.039). The AUC value of PCT for predicting bacterial infection was 0.618 (95% CI 0.524–0.711, *p* = 0.016). PCT level was significantly elevated in non-survivors (*n* = 13) compared with survivors (*n* = 133, *p* < 0.001) and independently associated with mortality (OR 1.075, 95% CI 1.003–1.152, *p* = 0.041). The AUC value of PCT for predicting mortality was 0.819 (95% CI 0.724–0.914, *p* < 0.001).

**Conclusion:** PCT concentrations at admission would be predictive of disease severity, secondary bacterial infection and mortality in patients with HFRS caused by Hantaan virus.

## Introduction

Hantaviruses, a group of enveloped single-stranded negative RNA viruses of the genus orthohantavirus in *Hantaviridae* family under *Bunyavirales* Order (Schmaljohn and Dalrymple, 1983; Schmaljohn et al., 1985; Adams et al., 2017), can cause hemorrhagic fever with renal syndrome (HFRS) primarily in Eurasia, and hantavirus cardiopulmonary syndrome in the Americas (Lee et al., 1978; Peters et al., 1999; Avsic-Zupanc et al., 2015). There are at least four HFRS-causing hantaviruses: Hantaan, Seoul, Puumala, and Dobrava viruses (Schmaljohn and Hjelle, 1997; Hart and Bennett, 1999). Clinically, typical HFRS occurs in five consecutive phases: febrile, hypotensive, oliguric, polyuric and convalescent (Peters et al., 1999; Vaheri et al., 2013). Pathophysiologically, endothelial dysfunction and increased vascular permeability are the fundamental characteristic of HFRS (Vaheri et al., 2013; Hepojoki et al., 2014) and the major explanation of the symptoms, such as hypotension, and features, such as the extravasation of fluid from intravascular to extravascular spaces in HFRS.(Kanerva et al., 1998; Vaheri et al., 2013; Ermonval et al., 2016).

Hantaan virus and Seoul virus, which are associated with a severe form and a mild form of HFRS, respectively, are the major etiologies of HFRS in China (Song, 1999; Zhang et al., 2010). In spite of increasing implementation of preventive measures including vaccination, HFRS remains a significant infectious disease with relatively high morbidity and mortality in China (Huang et al., 2012; Zhang et al., 2014), with approximately 10,000 cases of HFRS being reported annually (Jonsson et al., 2010; Huang et al., 2012; Zhang et al., 2014). Clinically, parameters which may be used as references of clinical significance for the severity assessment and prognosis prediction are necessities for improving the management of HFRS patients.

Procalcitonin (PCT), a precursor hormone of calcitonin, may be secreted by different cells in multiple organs when the body is stimulated by an inflammatory response, especially bacterial infection (Nishikura, 1999; Linscheid et al., 2003). Many studies have shown that PCT has an excellent predictive ability for sepsis (Arora et al., 2017; Nishikawa et al., 2017) and increased PCT is indicative of a risk of bacteremia in patients with acute fever (Kim et al., 2011). Notably, in hantavirus infections, an increased serum PCT level has been found in patients with acute nephropathia epidemica (NE) caused by Puumala virus (Jereb et al., 2011) although no association between PCT levels and severity of disease was observed (Latus et al., 2015a). PCT determined on admission to the hospital was also not shown to be able to predict the severity of acute kidney injury (AKI) in NE (Bunz et al., 2015). Differentially, patients with HFRS caused by Dobrava virus appeared to have a higher PCT level than those with Puumala virus infections (Jereb et al., 2011). However, few studies have investigated the usefulness of PCT for predicting the disease severity and prognosis in patients with the severe form HFRS caused by Hantaan virus. Therefore, the aim of this study was to analyse PCT levels in HFRS patients from Shaanxi, one of the high-endemic areas of the severe form HFSR caused by Hantaan virus in northwest China.(Huang et al., 2012; Ma et al., 2014; Tian et al., 2017).

## Methods

### Study population

Data from HFRS patients admitted from January, 2011 to December, 2016 in the First Affiliated Hospital of Xi'an Jiaotong University, a large tertiary-care hospital located in Xi'an, Shaanxi, northwest China, were retrospectively collected. Patients who are pregnancy or younger than 18 years, diagnosed with AKI other than HFRS, chronic kidney disease, liver diseases, cancer or hemopathy and used anticoagulants prior to admission to hospital were all excluded from the study. The data from 146 adult HFRS patients were included in the analysis of the study. The disease severity in the patients was classified into mild, medium, severe and gravis clinical types according to the diagnostic criteria from the *Prevention and Treatment Strategy of HFRS* by the Ministry of Health, People's Republic of China as described elsewhere (Liu et al., 2008). Clinically, 35 patients were classified as mild type, 37 medium type, 22 severe type, and 52 gravis type.

This retrospective study was conducted in accordance with the Declaration of Helsinki and the protocol was approved by the Ethics Committee of the First Affiliated Hospital of Xi'an Jiaotong University. Informed consent was not obtained from the patients as the patient records and information were anonymized and de-identified prior to the study.

### Determination of procalcitonin, WBC and platelet

PCT was measured using VIDAS® B•R•A•H•M•S PCT™ assay (bioMérieux, S.A., Marcy l'Etoile, France). The low detection limit of this method is 0.05 ng/ml and the reference value of normal range is <0.5 ng/ml. WBC and platelet were determined by sysmex XE-2100 fully automatic hematology analyzer (TOA Medical Electronics, Kobe, Japan). The normal reference ranges of WBC and platelet are 3.5–9.5 × 10^9^ cells/L and 125–350 × 10^9^ cells/L, respectively. The tests were performed at the central lab of the hospital.

### Data management and laboratory parameters

Demographic and clinical data collected from the HFRS patients included the patient's age, sex, max temperature, admission day after fever onset, blood pressure at the time of the assessment, cigarette and alcohol consumption, hospital stay, blood transfusion, continuous renal replacement therapy (CRRT), comorbidities (hypertension, diabetes mellitus, and coronary heart disease), and HFRS-related complications including hemorrhage, secondary bacterial infection, hepatic injury, sepsis, multiple organ dysfunction syndrome (MODS), and arrhythmia. Hemorrhage was defined as presentation of signs of hemorrhage (pulmonary hemorrhage, melena, hematemesis, hematuria, diffuse ecchymosis, and hematoma) during hospitalization. The diagnosis of bacterial infection was confirmed by clinical manifestation combined with serological and radiologic evidence, the presence of pathogenic bacteria by bacteriology and findings of infection features by imaginology examination. Sepsis and MODS were defined according to the criteria determined by Vincent et al. (2006) and the American-College of Chest Physicians Society of Critical Care Medicine Consensus Conference definition (Bone et al., 1992), respectively. Variables collected at hospital admission included serum PCT, white blood cell counts (WBC), platelet (PLT), neutrophil percentage, and lymphocyte percentage.

For the analysis of factors associated with disease severity, the patients were classified into two groups, with the patients with mild and medium types being designated as the mild group and the patients with severe and gravis types being included in the severe group. The factors potentially associated with secondary bacterial infection or the survival of the patients were analyzed according to the development of bacterial infection in the patients during hospitalization and the prognosis of the patients.

### Statistical analysis

Statistical analysis was performed using SPSS 20.0 software (SPSS Inc., Chicago, IL, USA). Log transformation was used to make data conform to normality and reduce the variability of data, especially in data sets that include outlying observations. Categorical data were presented as numbers and percentages and were analyzed using the Fisher's Exact or Pearson‘s Chi-square test where appropriate. Continuous, normally distributed data were described using mean and standard deviation and analyzed using Student's *t*-test. Non-parametric data were described using median and interquartile range (IQR) and analyzed using Mann-Whitney U test as appropriate. Results with a *p*-value of <0.05 were deemed as statistically significant. Variables found to be significantly associated with disease severity or death were tested in a logistic regression model for their potential to predict the corresponding outcome. Considering the effects that multicollinearity may have on regression analyses and subsequent results, variance inflation factor (VIF) was used to detect multicollinearity among variables. Variables with statistically significant results in the univariate analyses and without multicollinearity were included in multivariate logistic regression analyses for independent variables. Predictive values of variables for disease severity, bacterial infection or prognosis were tested with receiver operating characteristic (ROC) curves and quantified by calculating the area under the ROC curve (AUC) and the 95% confidence interval (CI).

## Results

### PCT association with disease severity

Of the 146 patients (109 males and 37 females; mean age 44.98 ± 16.08 years), 72 patients were included in the mild group, and 74 patients were included in the severe group. The mortality rate in the severe patient group was significantly higher than in the mild patient group (*p* = 0.002). With regard to HFRS-related complications, patients in the severe group had higher frequencies of hemorrhage, hepatic injury, sepsis, and MODS than those in the mild group (*p* = 0.001, *p* = 0.032, *p* = 0.040, and *p* = 0.012, respectively). The hospital stay was significantly longer in the severe group patients than in the mild group (*p* < 0.001). Patients in the severe group had more frequent blood transfusion and CRRT than those in the mild group (both *p* < 0.001). Patients in the severe group had significantly higher WBC and lower PLT levels (*p* < 0.001 and *p* = 0.007, respectively). The median serum PCT level in the 146 HFRS patients was 1.53 ng/ml (range 0.03–62.91 ng/ml), which is higher than the reference value of normal range (<0.5 ng/ml). The PCT levels in the severe group patients were significantly higher than those in the mild group patients (*p* < 0.001, Table 1).

**Table 1:** Demographics and clinical and laboratory data at admission in the patients with HFRS of different clinical types.

**Variables**	**Mild (*n* = 72)**	**Severe (*n* = 74)**	***P-*value**
Male, *n* (%)	49 (68.1)	60 (81.1)	0.070
Age, years	43.79 ± 16.71	46.13 ± 15.46	0.380
Max temperature, °C ±	38.9 ± 0.77	39.05 ± 0.73	0.280
Admitted days after fever, days	5.92 ± 4.08	6.14 ± 2.94	0.711
SBP, mmHg	120 (17.5)	118 (23.5)	0.638
DBP, mmHg	79 (14)	78 (19.25)	0.508
Smoking, *n* (%)	32 (44.4)	39 (52.7)	0.318
Alcohol consumption, *n* (%)	29 (40.3)	37 (50)	0.238
**COMORBIDITY**			
Hypertension, *n* (%)	11 (15.3)	12 (16.2)	0.876
Diabetes mellitus, *n* (%)	6 (8.3)	1 (1.4)	0.113
Coronary heart disease, *n* (%)	3 (4.2)	2 (2.7)	0.975
**HFRS-RELATED COMPLICATION**			
Hemorrhage, *n* (%)	31 (43.1)	52 (70.3)	0.001
Bacterial infection, *n* (%)	42 (58.3)	45 (60.8)	0.760
Hepatic injury, *n* (%)	32 (44.4)	46 (62.2)	0.032
Sepsis, *n* (%)	0	6 (8.1)	0.040
MODS, *n* (%)	0	8 (10.8)	0.012
Arrhythmia, *n* (%)	2 (2.8)	7 (9.5)	0.182
Blood transfusion, n (%)	11 (15.3)	44 (59.5)	<0.001
CRRT, n (%)	0	39 (52.7)	<0.001
Hospital stay, days	10 (1-27)	13 (3–47)	<0.001
Number of deaths, *n* (%)	1 (1.4)	12 (16.2)	0.002
**PARAMETERS**			
WBC, × 10^9^ cells/L	8.07 ± 6.70	11.81 ± 11.23	< 0.001
PLT, × 10^9^ cells/L	100.82 ± 79.83	66.74 ± 70.85	0.007
Neutrophils percentage, (%)	65.60 ± 16.29	69.09 ± 13.76	0.165
Lymphocytes percentage, (%)	23.99 ± 12.98	20.76 ± 11.13	0.109
PCT, (ng/ml)	0.81 (0.03-23.39)	2.74 (0.08–62.91)	<0.001
Lg PCT	−0.11 ± 0.66	0.44 ± 0.62	<0.001

*SBP, systolic blood pressure; DBP, diastolic blood pressure; MODS, multiple organ dysfunction syndrome; CRRT, continuous renal replacement therapy; WBC, white blood cell count; PLT, platelet; Lg PCT, procalcitonin after log10 transformation*.

In the 59 HFRS patents without secondary bacterial infection (30 patients in mild group and 29 patients in severe group), the median serum PCT level in the severe group patients [1.20 ng/ml (range 0.08–25.00 ng/ml)] was higher than that in the mild group patients [0.79 ng/ml (range 0.05–23.39 ng/ml)] although the difference was not statistically significant (*p* = 0.085). In the 140 HFRS patients without sepsis (72 patients in mild group and 68 patients in severe group), the median serum PCT levels in the severe group patients [2.49 ng/ml (range 0.08–62.91 ng/ml)] were statistically higher than those in the mild group patients [0.81 ng/ml (range 0.03–23.39 ng/ml, *p* < 0.001)]. Alternatively, the mean (± standard deviation, SD) serum PCT levels in the severe group patients (5.87 ± 9.36 ng/ml) were also statistically higher than those in the mild group patients (2.40 ± 4.56 ng/ml, *p* = 0.006). In the 87 HFRS patients with bacterial infection (42 patients in mild group and 45 patients in severe group), the median serum PCT level in the severe group patients [3.73 ng/ml (range 0.28–62.91 ng/ml)] was higher than that in the mild group patients [0.96 ng/ml (range 0.03–15.44 ng/ml), *p* < 0.001].

Multivariate analysis for the entire cohort revealed that PCT, WBC and hemorrhage were independent factors associated with the severity of HFRS (OR 2.544, 95% CI 1.330–4.868, *p* = 0.005; OR 1.082, 95% CI 1.014–1.155, *p* = 0.017 and OR 2.149, 95% CI 1.004–4.599, *p* = 0.049; respectively, Table 2).

**Table 2:** Independent risk factors for the severity of HFRS.

	**B**	**SE**	**Wald**	***P-*value**	**OR**	**95% CI for OR**	
						**Lower**	**Upper**
Lg PCT at admission	0.934	0.331	7.954	0.005	2.544	1.330	4.868
WBC at admission	0.079	0.033	5.690	0.017	1.082	1.014	1.155
Hemorrhage	0.765	0.388	3.879	0.049	2.149	1.004	4.599
Constant	−2.223	0.716	9.632	0.002	0.108		

*Forward: LR; B, independent variable coefficient; SE, standard error; OR, odds ratio; CI, confidence interval; WBC, white blood cell count; Lg PCT, procalcitonin after log10 transformation*.

**Figure 1:**
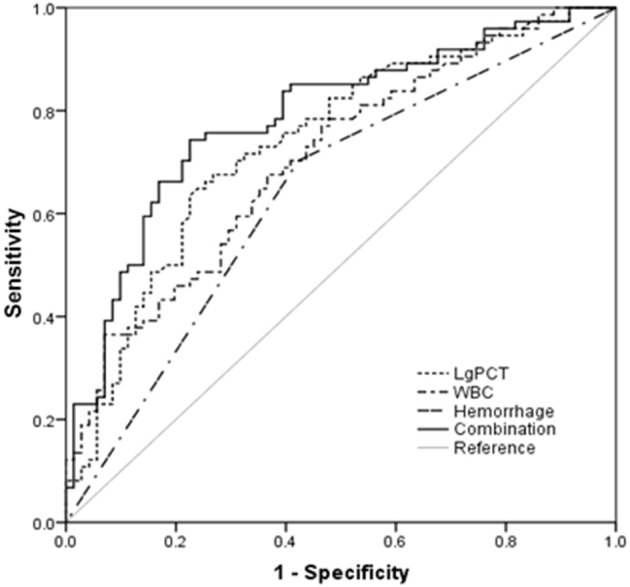
Procalcitonin (PCT) and white blood cell (WBC) at admission and the occurrence of hemorrhage in predicting the severity of hemorrhagic fever with renal syndrome (HFRS) by receiver operating characteristic curve (ROC) analysis.

The AUC values of PCT, WBC and hemorrhage for predicting the severity of HFRS were 0.738 (95% CI 0.657–0.820, *p* < 0.001), 0.706 (95% CI 0.623–0.789, *p* < 0.001), and 0.640 (95% CI 0.549–0.731, *p* = 0.004, Figure 1), respectively. By combining these 3 factors, the AUC value rose to 0.785, and the sensitivity and specificity were 74.0 and 78.0%, respectively (Table 3, Figure 1).

**Table 3:** Predictive values of parameters for the severity of HFRS.

**Variables**	**AUC**	***p* value**	**Cut-off value**	**Sensitivity**	**Specificity**	**95% CI for AUC**	
						**Lower**	**Upper**
Lg PCT at admission	0.738	<0.001	0.26	0.65	0.76	0.657	0.820
WBC at admission	0.706	<0.001	9.30	0.68	0.63	0.623	0.789
Hemorrhage	0.640	0.004	–	–	–	0.549	0.731
Combination^a^	0.785	<0.001	0.49^b^	0.74	0.78	0.710	0.861

*AUC, area under the receiver operating characteristic (ROC) curve; P-value for calculated AUC in predicting severity; CI, confidence interval; Sensitivity, specificity and 95% CI are all presented as percentages; WBC, white blood counts; LgPCT, procalcitonin after log10 transformation*.

a *LgPCT, WBC, and the occurrence of hemorrhage in combination*.

b *Probability value of the combination was analyzed by logistic regression. The regression coefficients of these three parameters were used to set up a logit model for the progression of HFRS as follows: Logit(P|y = severe type) = −2.223+0.079LgPCT +0.934WBC+0.765Hemorrhage*.

### PCT association with bacterial infection

Of the 146 patients, 87 patients were diagnosed as having secondary bacterial infection. The incidence of bacterial infection in the patients during the hospitalization was 59.6%. Neutrophil and lymphocyte percentages were significantly different between patients with bacterial infection and those without (*p* = 0.031 and *p* = 0.008, respectively, Table 4). PCT levels in patients with bacterial infection were significantly higher than in those without bacterial infection (*p* = 0.037, Table 4). The median serum PCT level in the 87 HFRS patients with bacterial infection [2.00 ng/ml (range 0.03–62.91 ng/ml)] was significantly higher than that in the 59 HFRS patients without bacterial infection [0.94 ng/ml (range 0.05–25 ng/ml), *p* = 0.016].

**Table 4:** Demographics, clinical data and laboratory perameters at admission in HFRS patients with and without bacterial infection.

**Variables**	**Non-bacterial infection (*n* = 59)**	**Bacterial infection (*n* = 87)**	***P-*value**
Male, *n* (%)	46 (42.2)	63 (57.8)	0.449
Age, years	41.93 ± 14.53	47.05 ± 16.82	0.059
Max temperature, °C	38.86 ± 0.75	39.07 ± 0.74	0.097
Admitted days after fever	5.80 ± 2.66	6.18 ± 4.04	0.518
SBP, mmHg	121.80 ± 20.24	119.78 ± 17.10	0.518
DBP, mmHg	78.03 ± 13.48	78.00 ± 14.87	0.989
Smoking, *n* (%)	32(54.2)	39 (44.8)	0.264
Alcohol consumption, *n* (%)	30 (50.8)	36 (41.4)	0.259
**COMORBIDITY**			
Hypertension, *n* (%)	10 (16.9)	13 (14.9)	0.744
Diabetes mellitusm, *n* (%)	2 (3.4)	5 (5.7)	0.513
Coronary heart disease, *n* (%)	1 (1.7)	4 (4.6)	0.344
**HFRS-RELATED COMPLICATION**			
Hemorrhage, *n* (%)	33 (55.9)	50 (57.5)	0.854
Hepatic injury, *n* (%)	30 (50.8)	48 (55.2)	0.607
Sepsis, *n* (%)	1 (1.7)	5 (5.7)	0.226
MODS, *n* (%)	2 (3.4)	6 (6.9)	0.361
Arrhythmia, *n* (%)	2 (3.4)	7 (8.0)	0.251
Blood transfusion, *n* (%)	18 (30.5)	37 (42.5)	0.141
CRRT, *n* (%)	13 (22)	26 (29.9)	0.293
Hospital stay, days	12.32 ± 7.26	13.30 ± 7.06	0.419
Number of deaths, *n* (%)	2 (3.4)	11 (12.6)	0.054
**PARAMETERS**			
WBC, × 10^9^ cells/L	11.79 ± 8.32	12.06 ± 7.08	0.831
PLT, × 10^9^ cells/L	82.19 ± 74.46	84.28 ± 79.17	0.873
Neutrophils percentage, (%)	64.05 ± 13.01	69.56 ± 16.07	0.031
Lymphocytes percentage, (%)	25.62 ± 11.37	20.20 ± 12.24	0.008
PCT,(ng/ml)	0.94(0.05-25.00)	2.00 (0.03–62.91)	0.016
Lg PCT	0.02 ± 0.68	0.27 ± 0.69	0.037

*SBP, systolic blood pressure; DBP, diastolic blood pressure; MODS, multiple organ dysfunction syndrome; CRRT, continuous renal replacement therapy; WBC, white blood cell counts; PLT, platelet; PCT, procalcitonin; Lg PCT, procalcitonin after log10 transformationn*.

Univariate logistic regression analysis showed that PCT, neutrophil percentage, and lymphocyte percentage are associated with bacterial infection (OR 1.685, 95% CI 1.026–2.768, *p* = 0.039; OR 1.025, 95% CI 1.002–1.049, *p* = 0.035 and OR 0.963, 95% CI 0.936–0.991, *p* = 0.010; respectively). Multivariate logistic regression analysis was not performed because of the multicollinearity among PCT, neutrophils percentage and lymphocyte percentage (VIF = 3.829) which may incur false results in multivariate logistic regression.

**Figure 2:**
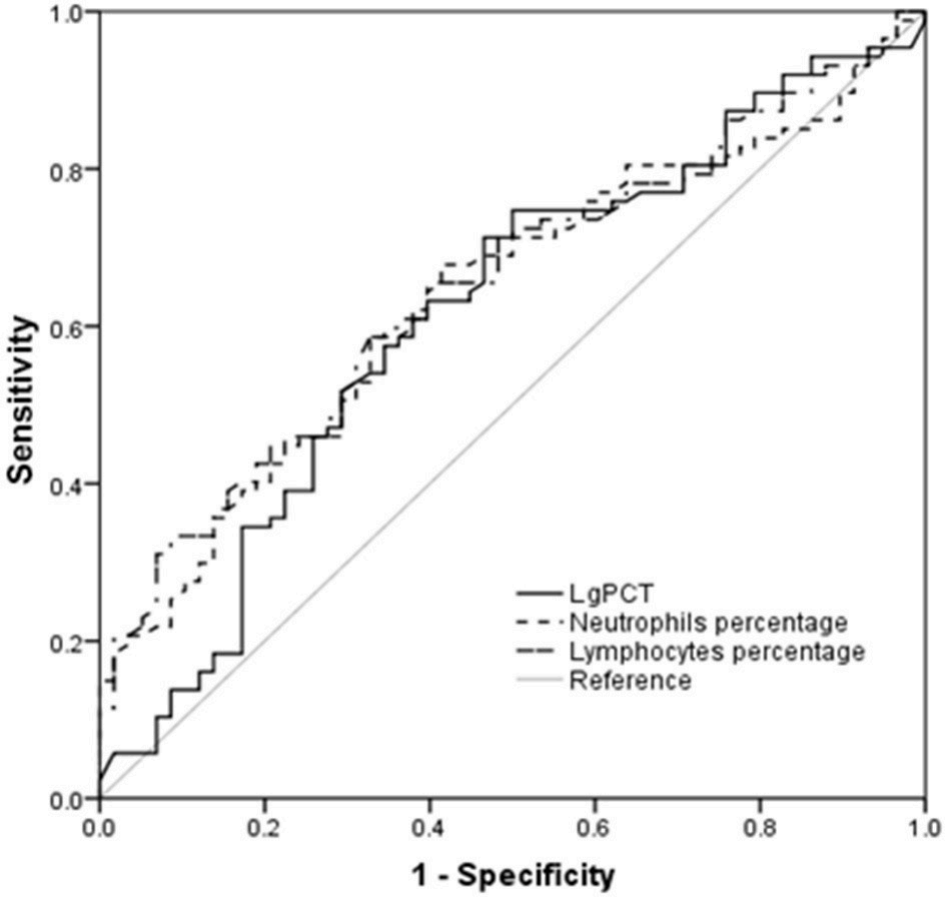
Procalcitonin (PCT) and the percentages of neutrophils and lymphocytes in predicting secondary bacterial infection in hemorrhagic fever with renal syndrome (HFRS) patients by receiver operating characteristic curve (ROC) analysis.

The AUC values of PCT, neutrophil percentage, and lymphocyte percentage for predicting bacterial infection were 0.618 (95% CI 0.524–0.711, *p* = 0.016), 0.639 (95% CI 0.550–0.729, *p* = 0.005) and 0.651 (95% CI 0.563–0.740, *p* = 0.002), respectively (Table 5, Figure 2).

**Table 5:** Predictive values of parameters for bacterial infection in HFRS patients.

**Variables at admission**	**AUC**	***P*-value**	**Cut-off value**	**Sensitivity**	**Specificity**	**95% CI for AUC**	
						**Lower**	**Upper**
Lg PCT at admission	0.618	0.016	0.00	0.71	0.54	0.524	0.711
Neutrophils percentage at admission	0.639	0.005	67.47	0.68	0.59	0.550	0.729
Lymphocytes percentage at admission	0.651	0.002	18.51	0.67	0.59	0.563	0.740

*AUC, area under the receiver operating characteristic (ROC) curve; CI, confidence interval; Lg PCT, procalcitonin after log10 transformation*.

### PCT association with patient survival

Of the 146 HFRS patients, 13 patients died of the disease, with a mortality rate of 8.9%. Non-survivors of the patients had older age and lower diastolic blood pressure (DBP) at hospital admission (*p* = 0.010 and *p* = 0.030, respectively, Table 6). The presence of hemorrhage, MODS and sepsis was significantly higher in non-survivors compared with survivors (*p* = 0.007, *p* < 0.001 and *p* = 0.001, respectively, Table 6). Blood transfusion and CRRT were more frequently applied to non-survivors compared with survivors (*p* = 0.001 and *p* < 0.001, respectively, Table 6) although the non-survivors had a shorter hospital stay because of deaths during hospitalization (*p* = 0.042). Non-survivors had a significantly higher neutrophil percentage and lower lymphocyte percentage compared with survivors (*p* = 0.012 and *p* = 0.031, respectively, Table 6). The PCT levels in non-survivors were significantly elevated compared with survivors (*p* < 0.001, Table 6).

**Table 6:** Demographics, clinical data and laboratory perameter at admission in survivors and non-survivors of patients with HFRS.

**Variables**	**Survivors (*n* = 133)**	**Non-survivors (*n* = 13)**	***P*-value**
Male, *n* (%)	100 (75.2)	9 (69.2)	0.891
Age, years	43.92 ± 16.18	55.76 ± 10.28	0.010
Max temperature, °C	38.99 ± 0.75	38.91 ± 0.77	0.723
Admitted days after fever	5.99 ± 3.59	6.46 ± 3.07	0.645
SBP, mmHg	121.17 ± 17.94	114.69 ± 22.50	0.226
DBP, mmHg	78.81 ± 13.09	69.85 ± 22.36	0.030
Smoking, *n* (%)	63 (47.4)	8 (61.5)	0.329
Alcohol consumption, *n* (%)	59 (44.4)	7 (53.8)	0.512
**COMORBIDITY**			
Hypertension, *n* (%)	20 (15)	3 (23.1)	0.718
Diabetes mellitusm, *n* (%)	5 (3.8)	2 (15.4)	0.119
Coronary heart disease, *n* (%)	4 (3)	1 (7.7)	0.377
**HFRS-RELATED COMPLICATION**			
Hemorrhage, *n* (%)	71 (53.4)	12 (92.3)	0.007
Bacterial infection, *n* (%)	76 (57.1)	11 (84.6)	0.054
Hepatic injury, *n* (%)	68 (51.1)	10 (76.9)	0.075
Sepsis, *n* (%)	2 (1.5)	4 (30.8)	0.001
MODS, *n* (%)	2 (1.5)	6 (46.2)	<0.001
Arrhythmia, *n* (%)	9 (6.8)	0	1.000
Blood transfusion, n (%)	44 (33.1)	11 (84.6)	0.001
CRRT, n (%)	28 (21.1)	11 (84.6)	<0.001
Hospital stay, days	13.28 ± 7.11	9.08 ± 6.47	0.042
**LABORATORYPARAMETERS**			
WBC, × 10^9^ cells/L	11.78 ± 7.66	13.66 ± 6.79	0.395
PLT, × 10^9^ cells/L	86.23 ± 79.07	54.92 ± 44.68	0.163
Neutrophils percentage, (%)	66.38 ± 15.20	77.34 ± 10.16	0.012
Lymphocytes percentage, (%)	1.72 (2.86)	1.69 (1.34)	0.031
PCT, (ng/ml)	1.22 (0.03-62.91)	7.65 (1.06–58.53)	<0.001
Lg PCT	0.10 ± 0.68	0.87 ± 0.48	<0.001

*SBP, systolic blood pressure; DBP, diastolic blood pressure; MODS, multiple organ dysfunction syndrome; CRRT, continuous renal replacement therapy; WBC, white blood cell count; PLT, platelet; Lg PCT, procalcitonin after log10 transformation*.

In multivariate analysis after adjusting for other clinical parameters identified in univariate analysis, PCT levels, neutrophil percentage and MODS were shown to be independent factors associated with mortality in HFRS patients (OR 1.075, 95% CI 1.003–1.152, *p* = 0.041; OR 32.151, 95% CI 4.499–229.749, *p* = 0 .001 and OR 7.302, 95% CI 1.661–32.105, *p* = 0.009; respectively, Table 7).

**Table 7:** Independent risk factors associated with mortality in patients with HFRS.

**Variables**	**B**	**SE**	**Wald**	***P-*value**	**OR**	**95% CI for OR**	
						**Lower**	**Upper**
Lg PCT at admission	0.072	0.035	4.176	0.041	1.075	1.003	1.152
Neutrophil percentage at admission	3.470	1.003	11.963	0.001	32.151	4.499	229.749
MODS	1.988	0.756	6.924	0.009	7.302	1.661	32.105
Constant	−12.586	3.266	14.846	<0.001			

*Forward: LR; B, independent variable coefficient; SE, standard error; OR, odds ratio; CI, confidence interval, Lg PCT, procalcitonin after log10 transformation; MODS, multiple organ dysfunction syndrome*.

**Figure 3:**
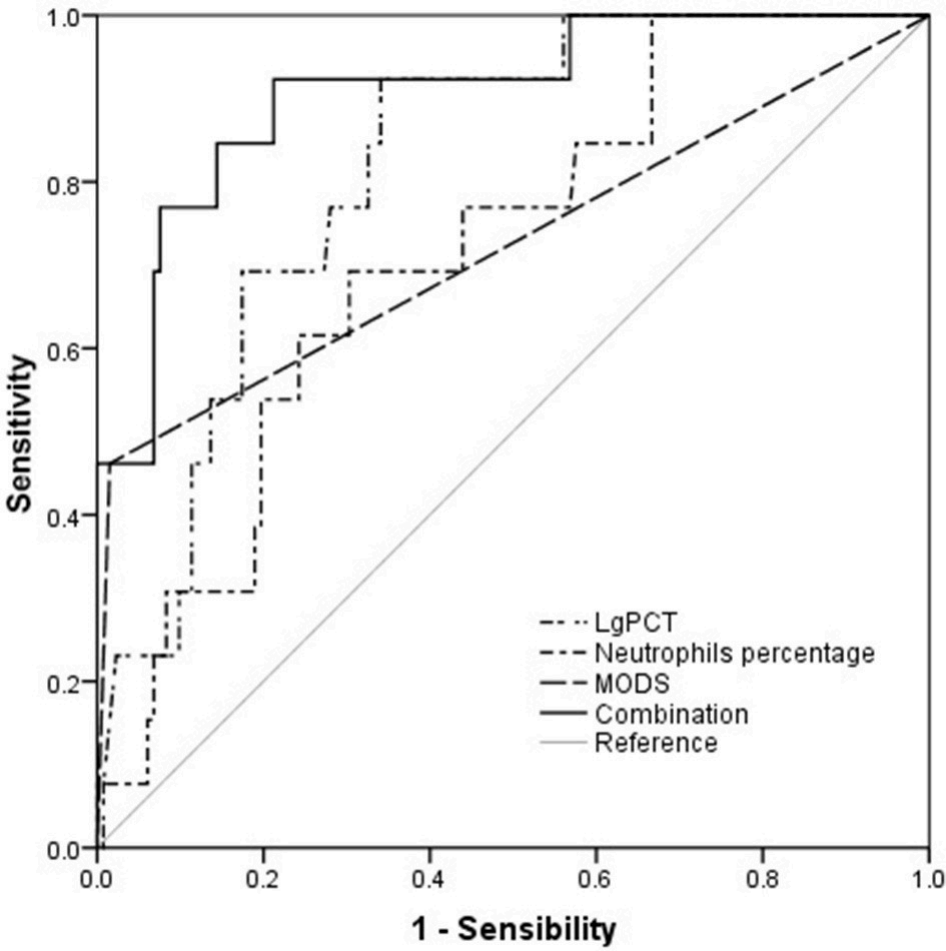
Procalcitonin (PCT) and the percentages of neutrophils at admission and the occurrence of multiple organ dysfunction syndrome (MODS) in predicting the prognosis of hemorrhagic fever with renal syndrome (HFRS) patients by receiver operating characteristic curve (ROC) analysis.

The AUC values of PCT, neutrophil percentage and MODS for predicting mortality were 0.819 (95% CI 0.724–0.914, *p* < 0.001), 0.716 (95% CI 0.586–0.847, *p* = 0.010) and 0.723 (95% CI 0.544–0.903, *p* = 0.008), respectively (Table 8, Figure 3). By combining these 3 factors, the AUC value rose to 0.907 (95% CI 0.821–0.993, *p* < 0.001) with a sensitivity of 92.0% and a specificity of 79.0%, respectively (Table 8, Figure 3).

**Table 8:** Predictive values of parameters for mortality of HFRS patients.

**Variables**	**AUC**	***P-*value**	**Cut-off value**	**Sensitivity**	**Specificity**	**95% CI for AUC**	
						**Lower**	**Upper**
Lg PCT at admission	0.819	<0.001	0.36	0.92	0.66	0.724	0.914
Neutrophils percentage at admission	0.716	0.010	73.60	0.69	0.70	0.586	0.847
MODS	0.723	0.008	–	–	–	0.544	0.903
Combination^a^	0.907	<0.001	0.06^b^	0.92	0.79	0.821	0.993

*AUC, area under the receiver operating characteristic (ROC) curve; CI, confidence interval; MODS, multiple organ dysfunction syndrome*.

a *Lg PCT, Neutrophil percentage, and the occurrence of MODS in combination*.

b *Probability value of the combination was analyzed by logistic regression. The regression coefficients of these three parameters were used to set up a logit model of death for critical HFRS patients as follows: Logit(P|y = death) = −12.586+0.072LgPCT+3.470neutrophil percentage+1.988 MODS*.

## Discussion

This study, for the first time to our knowledge, analyzed the changes of PCT levels in patients with HFRS caused by Hantaan virus. The results showed that serum PCT levels were elevated and associated with the disease severity, secondary bacterial infection and mortality in the patients.

This study, consistent with the findings in Puumala virus and Dobrava virus infection (Jereb et al., 2011; Bunz et al., 2015; Latus et al., 2015a), showed significantly elevated PCT levels in Hantaan virus-associated HFRS. It is well known that endotoxin may induce PCT in human (Dandona et al., 1994). Moreover, tumor necrosis factor (TNF)-α and interleukin (IL)-6 can induce PCT production in the absence of endotoxin (Nijsten et al., 2000). Increased concentrations of TNF-α and IL-6 have been demonstrated in HFRS patients (Linderholm et al., 1996). Therefore, the increased PCT levels documented in HFRS patients may be resulted from the stimuli by elevated TNF-α and IL-6 even in the absence of bacterial infection.

Although it was not applicable for the present study to compare PCT levels between individuals with Hantaan virus infection and those with other hantaviruses such as Puumala virus and Dobrava virus, PCT levels in patients with Hantaan virus-associated HFRS appeared to be higher than those in Puumala virus. The median serum PCT level in HFRS patients without bacterial infection was 0.94 ng/ml (range 0.05–25 ng/ml) in the present study. A previous study showed that patients with HFRS caused by Dobrava virus seemed to have a higher PCT level [0.74 μg/L (range 0.09–2.83 μg/L)] than those with NE caused by Puumala virus [0.50 μg/L (range 0.10–11.71 μg/L)] (Kim et al., 2011). Because Dobrava virus and Hantaan virus cause more severe clinical diseases compared with Puumala virus, it is suggested that different hantaviruses cause diseases of different clinical severity, resulting in the variations of PCT levels. A study showed that there were notable differences in viral load and antibody and cytokine response dynamics between Dobrava and Puumala infections, and these may be reflected in differing disease severities and clinical outcomes (Korva et al., 2013). Another study showed that patient with Dobrava-Belgrade virus infection had a prolonged clinical course and a late and enhanced mobilization of cytokines compared with patient with Puumala virus infection and these differences in cytokine deregulation may contribute to the variations in the clinical course (Krautkrämer et al., 2016). It is possible that the higher PCT levels in patients with Hantaan virus infection may be at least partly associated with the enhanced responses to the virus, especially the higher magnitude of cytokine responses in the patients.

Elevated PCT level is documented to be especially indicative of bacterial infection (Nishikura, 1999; Linscheid et al., 2003; Kim et al., 2011; Arora et al., 2017; Nishikawa et al., 2017) and PCT has been increasingly acknowledged to be a major biomarker of bacterial infection. Serum concentration of the PCT is also indicated to be a good marker in differentiating infectious from noninfectious causes of inflammation (Assicot et al., 1993). However, this study and other studies (Jereb et al., 2011; Bunz et al., 2015; Latus et al., 2015a) demonstrated the common presence of higher PCT level in infection of hantaviruses. It is suggested that PCT level may be of less clinically differential quality between infections of hantaviruses and bacteria and caution should be taken in the interpretation of PCT levels when hantavirus infection is suspected. Moreover, a study showed that PCT failed to predict bacteremia in systemic inflammatory response syndrome (SIRS) patients (Hoenigl et al., 2014) although it can accurately differentiate culture-negative sepsis from noninfectious SIRS (Anand et al., 2015). A study in trauma patients showed that PCT was related to inflammation caused by injury but not to infection (Mimoz et al., 1998). PCT is also indicated to be unable to predict between bacterial and viral meningitis (Sanaei Dashti et al., 2017).

With respect to the disease severity, this study showed that elevated PCT level, along with WBC and hemorrhage, was significantly associated with the disease severity in HFRS patients with Hantaan virus infection. It is acceptable that WBC count and hemorrhage are suggestive of the disease severity in that WBC count is a commonly used parameter of inflammation and hemorrhage is a prominent feature of the severe form HFRS. The leukocyte at admission was predictive of the subsequent development of oliguric acute renal failure in HFRS (Kim et al., 2007). In intracerebral hemorrhage patients, elevated PCT levels at admission were shown to be independently associated with unfavorable clinical outcome (He et al., 2017). This study demonstrated that PCT level is not only an independent factor associated with the severity of HFRS but also a parameter with slightly higher AUC value than WBC and hemorrhage for predicting the severity of HFRS. In patients with NE caused by Puumala virus and HFRS caused by Dobrava virus, no association between PCT levels and severity of disease was observed (Jereb et al., 2011; Bunz et al., 2015; Latus et al., 2015a). However, these studies were performed in relatively small number of patients and this may affect the results of the analysis. The comparison of PCT levels between patients with HFRS caused by Dobrava virus and those with Puumala virus infections (Jereb et al., 2011) had a smaller patient number but it indicated a higher PCT level in HFRS caused by Dobrava virus. The present study analyzed PCT levels in a relatively larger number of patients with severe form HFRS which may manifest severe inflammation. PCT concentrations may be proportional to the inflammatory stimulus. Elevated PCT levels represent more pronounced systemic inflammation, which might be associated with a more severe disease course. The association of PCT level with disease severity in diseases caused by different hantaviruses may be virus type and disease severity specific. Of course, further studies are needed to clarify the relationship between PCT levels and disease severity in various hantavirus infections.

Regarding secondary bacterial infection in HFRS patients, this study showed that PCT level, in addition to higher percentage of neutrophils, and lower percentage of lymphocytes, was a significant factor associated with bacterial infection. The significance of neutrophil and lymphocyte percentages has been widely accepted as indicators of bacterial infection. Consistent with the result in this study, a previous study in hantavirus infections showed that increased serum PCT level was found to exhibit overlapping results between viral and severe bacterial infections (Jereb et al., 2011). Notably, the value of PCT for predicting bacterial infection in HFRS patients appeared to be low in the current study. This is possibly related to the intrinsically increased PCT caused by the primary disease *per se* since HFRS patients without bacterial infection had a higher serum PCT level [0.94 ng/ml (range 0.05–25 ng/ml)] than the normal reference value (<0.5 ng/ml) and this may compromise the performance of PCT in identifying bacterial infections in this specific disease condition. Therefore, different thresholds of PCT should be applied in HFRS patients when secondary bacterial infection is suspected during the disease course.

With respect to the mortality of HFRS patients, this study showed that PCT, together with the percentage of neutrophils and MODS, was an independent factor and had a higher AUC value (0.819) than the percentage of neutrophils (0.716) and MODS (0.723) for predicting mortality of the patients. Severe systemic inflammation is a common characteristic of severe HFRS. Neutrophils, in addition to their contribution to chronic inflammatory conditions and adaptive immune responses, are major effectors of acute inflammatory reaction (Kolaczkowska and Kubes, 2013). Increased PCT level and neutrophil percentage represent severe systemic inflammation, which may potentially contribute to a more severe disease course and increased mortality. MODS may occur in severe HFRS patients and contribute to the increased mortality of the patients (Zhao et al., 2009). PCT has been indicated to represent a sensitive and predictive indicator of severe MODS in injured patients (Wanner et al., 2000). Therefore, MODS is not only an independent factor of the mortality in HFRS patients but also a significant contributor of increased PCT levels in the patients. AKI is another prominent feature of HFRS. A study in patients after vascular surgery showed that renal function was a major determinant of PCT levels (Amour et al., 2008). Another study in critically ill patients showed a significant association of an elevated PCT on admission with the development of AKI in the non-septic patient (Jeeha et al., 2017). It is suggested that kidney injury in HFRS may be another contributor of the elevated PCT levels. Hemodynamical instability such as hypotension and even shock may develop in severe HFRS patients. A study in out-of-hospital cardiac arrest patients showed that elevated PCT was associated with hemodynamical instability and worsened long-term outcome (Pekkarinen et al., 2017). Therefore, the potential hemodynamical instability in HFRS patients may also relate to the elevation of PCT in HFRS patients. Taken together, multiple events such as severe SIRS, MODS, AKI and hemodynamical instability potentially presented in severe HFRS patients might promote the increase of PCT concentration associated with the increased mortality.

In conclusion, this study highlights the existence of a frequent association between HFRS and elevation of PCT in the absence of bacterial infection and the existence of overlapping results between Hantaan virus infection with and without bacterial infection. Importantly, higher PCT concentrations at hospital admission may be associated with disease severity, secondary bacterial infection and mortality in patients with HFRS caused by Hantaan virus. PCT level at admission in HFRS patients seems to aid early recognition of the disease severity and mortality with good performance and the secondary bacterial infection with moderate performance. These findings add novel information for the value of PCT application in clinical settings.

This study has some limitations. First, the retrospective nature of the study may affect the potency of the results. Second, the study is limited by the relatively small number of patients in a single medical center. Third, some parameters of inflammation such as elevated C-reactive protein levels, which have been shown to be associated with the severity in hantavirus-induced NE (Latus et al., 2015b), were not included in the study because of the non-routine detection in HFRS patients in our hospital. Fourth, the normal range of PCT from reference was used for the comparison of an elevated PCT levels in HFRS patients and no normal health control group was included for the comparison in the study. This may also potentially cause deviation to the results of the comparison of PCT levels between HFRS patients and normal individuals. Therefore, prospective studies with a large population of patients and normal health controls in multiple centers are needed to clarify the value of PCT in predicting the disease severity, bacterial infection and mortality of HFRS patients.

## Author contributions

XF and ZL contributed to conception and design of the research. XF, HD, JS, NL, QH, and XZ acquired data in the study. XF, NL, HD, JS, XZ, and QH analyzed data. XF and ZL drafted the paper, and all authors revised it critically, read and approved the final manuscript.

### Conflict of interest statement

The authors declare that the research was conducted in the absence of any commercial or financial relationships that could be construed as a potential conflict of interest.
